# Supplementary material for: Simplified Synthesis and Stability Assessment of Aflatoxin B1-Lysine and Aflatoxin G1-Lysine
Source: Toxins (Basel). 2022 Jan 14;14(1):56. doi: 10.3390/toxins14010056 (PMC8780103; doi:10.3390/toxins14010056)
Supplement: Supplementary file 1 [file toxins-14-00056-s001.zip › toxins-1537948-supplementary.pdf]

toxins

MDPI

Article

Simplified Synthesis and Stability Assessment of Aflatoxin B1-Lysine and Aflatoxin G1-Lysine

Justin B. Renaud [1,*], Jacob P. Walsh [1,2] and Mark W. Sumarah [1,2,*]

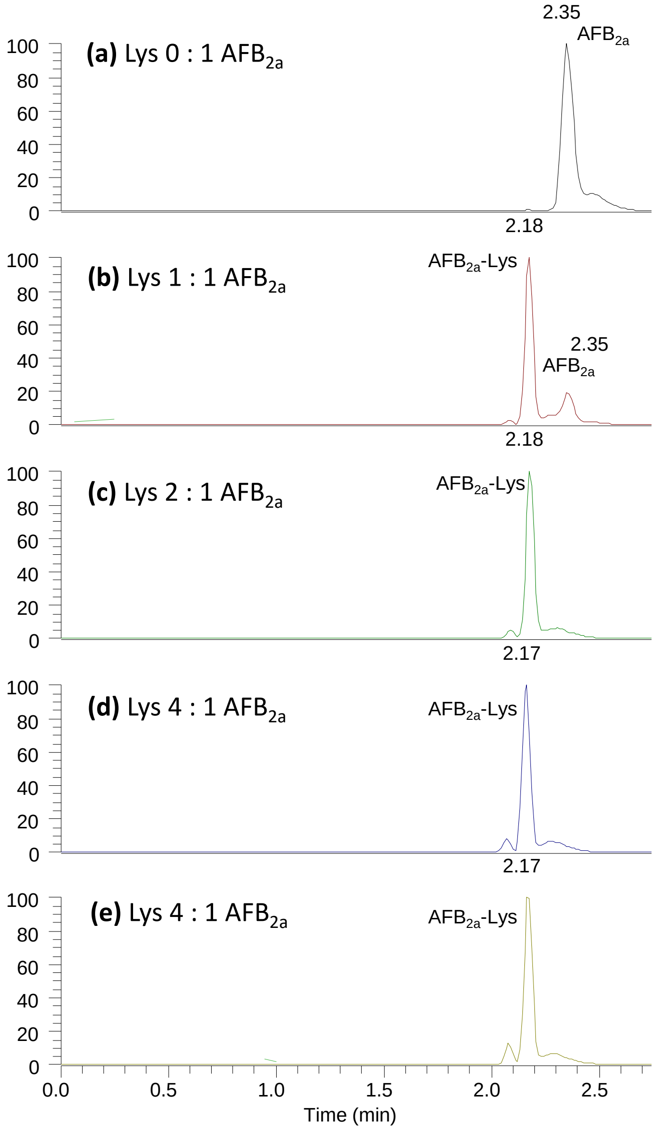

Figure S1. LC-MS/MS chromatogram of AFB_{2a} reacted with a molar ratio of 1 to (a) 0, (b)1, (c) 2, (d) 4, (e) 10. At a molar ratio above 2, no unreacted AFB_{2a} was detected.

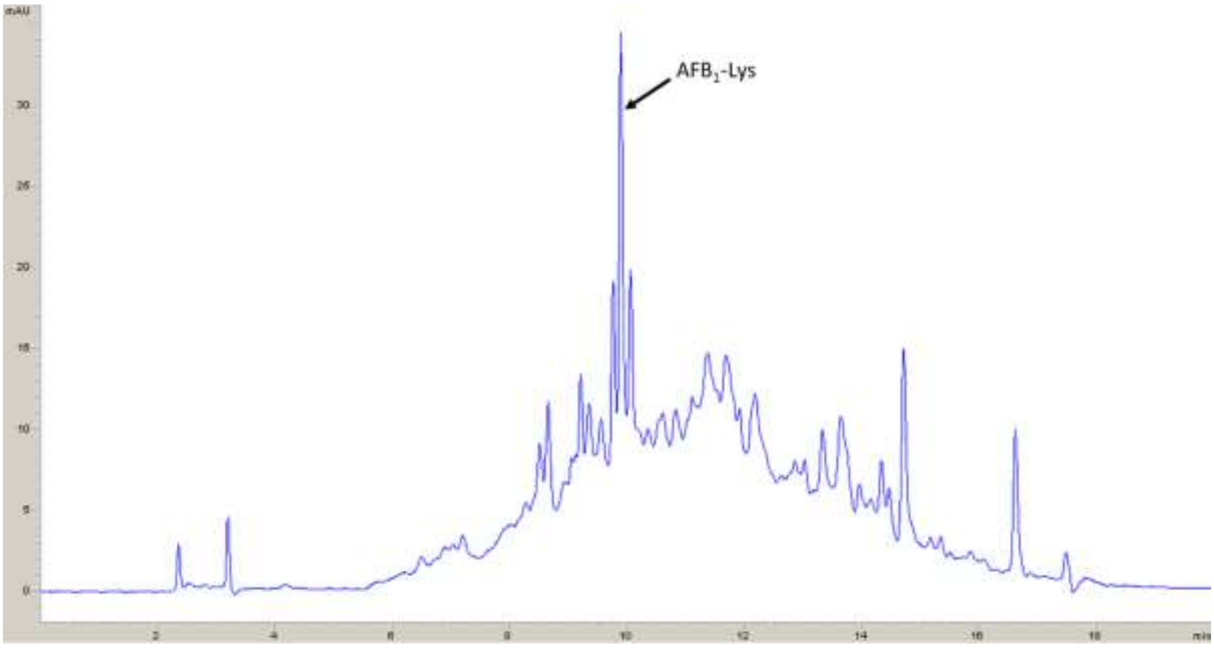

Figure S2. Chromatogram of isolation AFB_1-Lys was obtained using method described in section 4.5. AFB_1-Lys peak of interest is highlighted and was collected using time-based fraction collected. All fractions were screened by LC-MS to confirm identification. Chromatogram obtained at 399 nm referenced to 460 nm.

Table S1. Synthesis of Aflatoxin B1-lysine (AFB_1-lysine).

Note: this procedure can also be undertaken with a AFG_1 without modification. Similarly, different isotopically labelled lysines may also be used in place of the native lysine used herein.

1) Weigh out 1 mg of AFB_1 in an amber glass vial with a cap
a. NOTE: using plastic vials could result in the appearance of anti-static agents and plasticizers.
2) Dissolve 1 mg of AFB_1 in 400 µL of LC-MS grade acetonitrile.
a. Vortex for 15 s and sonicate in a water bath at 25 °C water bath for 3 minutes.
3) Add 500 µL of LC-MS grade H_2O and ensure that all the aflatoxin residue is fully dissolved.
4) Add 100 µL of 2M HCl into the solution, cap the vessel.
5) Place the glass vial at 45 °C and shaking at 300 rpm for up to 48 hours
a. Prior to proceeding to the next step, remove a 10 µL aliquot and transfer to 990 µL of 50% ACN. Check the reaction mixture by LC-MS for *m/z* 331 (AFB_{2a}) and *m/z* 313 (AFB_1) and ensure that the majority of AFB_1 has been converted. If not, continue incubation for 12-24 hours
6) Split the solution into 100 µg aliquots by transferring approximately 100µL into fresh amber HPLC vials
7) Carefully dry down the solution under nitrogen without heat.
a. NOTE: Adding heat could result in oxidation to occur
8) Add 200 µL of LC-MS grade acetonitrile to wash the dried residue and remove any residual acid.

a. NOTE: pH control during the coupling step is important and small amounts of acid can alter the pH of the dilute pH 10 buffer used
9) Resolubilize the dried residue (100 µg AFB_1 equiv.) in 100 µL of 0.05M sodium bicarbonate (pH 10.0).
10) Add 10 µL of lysine (19 mg/mL) dissolved in sodium bicarbonate (pH 10.0).
11) Incubate the solution at room temperature for at least 30 minutes.
a. Ensure that all the dried residue is mixing with the lysine solution by aspirating it with a pipette
b. Note: the intermediate AFB_{2a}-lysine is stable in this solution for up to 3 weeks when stored at 4 °C

FOR THE SYNTHESIS OF AFB_{2a}-lysine, END HERE

12) Prior to proceeding to the next step, remove a 10 µL aliquot and transfer to 990 µL of 50% ACN. Check the reaction mixture by LC-MS for *m/z* 441 (AFB_{2a}-lys) and *m/z* 331 (AFB_{2a}) and ensure that the majority of AFB_{2a} has coupled with the lysine. If not, add additional lysine to the solution.
13) Dry the solution down using a gentle stream of nitrogen with slight heat
a. NOTE: avoid high heat at this step as the pyrrole moiety can become easily oxidized.
14) Add 100 µL of MeOH to wash the dried AFB_{2a}-lysine residue and dry again under a gentle stream of nitrogen, without heat.
15) Resolubilize the AFB_{2a}-lysine residue (100 µg AFB_1 equiv.) in 200 µL of MeOH.
a. Vortex vigorously to ensure that the residue is completely resolubilized.

NOTE: Only continue with a 100 µg sub-aliquot to ensure that the concentration of H_2O_2 is optimal prior to adding H_2O_2 to all sub-aliquots

16) Dilute a stock solution of fresh H_2O_2 into water for a final concentration of 18.3% (v/v).
a. NOTE: Store the H_2O_2 solution on ice at all times.
17) Add 3 µL of the 18.3% to the AFB_{2a}-lysine methanol solution.
18) Incubate with shaking at room temperature for 1 hour.
19) Dry down the sample with a stream of nitrogen and gentle heat.
20) Resolubilize with 100 µL of 50% ACN, remove 10 µL and dilute into 990 µL of 50% ACN. Check the reaction mixture by LC-MS for *m/z* 457 (AFB_1-lys) and *m/z* 441 (AFB_{2a}-lys) and ensure that the majority of AFB_{2a}-lys has been oxidized. If greater than 50% of AFB_{2a}-lys remains, add an additional 2 µL of H_2O_2 and incubate for another hour. NOTE: Adding excess H_2O_2 could result in significant loss of product and it may be advisable to isolate the product that is there instead of attempting to generate more.
21) Dry down the solution with a gentle stream of nitrogen without heat in order to remove excess H_2O_2. The residue can be stored at -20 °C dry or reconstituted in 50% acetonitrile and stored at -20 °C for at least 3 weeks with limited degradation.
